# *OGDH* mediates α-ketoglutarate-induced follicular development and antioxidative response by interacting with CAT/SOD2

**DOI:** 10.1186/s40659-026-00688-9

**Published:** 2026-04-10

**Authors:** Enyuan Huang, Jingyu Zhou, Mengting Hu, Liuhong Zhang, Jiahao Shao, Meng Lv, Fen Miao, Yao Jiang, Nian Li, Jiaqi Li, Xiaolong Yuan

**Affiliations:** 1 State Key Laboratory of Swine and Poultry Breeding Industry, National Engineering Research Center for Breeding Swine Industry, Guangdong Provincial Key Laboratory of Agro-Animal Genomics and Molecular Breeding, College of Animal Science, South China Agricultural University, Guangzhou, 510642 Guangdong China; 2 Centre for Healthy Ageing, Health Futures Institute, Murdoch University, Murdoch, WA 6150 Australia; 3 School of Medical, Molecular and Forensic Sciences, Murdoch University, Murdoch, WA 6149 Australia; 4 Shenzhen Branch, Guangdong Laboratory for Lingnan Modern Agriculture, Genome Analysis Laboratory of the Ministry of Agriculture, Agricultural Genomics Institute at Shenzhen, Chinese Academy of Agricultural Sciences, Shenzhen, 518120 China

**Keywords:** Follicular disorder, Granulosa cells, Alpha-Ketoglutaric acid, *OGDH*

## Abstract

**Background:**

Follicular disorders, often driven by ROS-induced granulosa cell apoptosis, are a major cause of female infertility. While α-ketoglutarate (AKG), also known as 2-oxoglutarate, can improve follicular development, the underlying mechanisms remain unclear. Given that AKG is the primary substrate of oxoglutarate dehydrogenase (*OGDH*), this study aimed to investigate how *OGDH* mediates the protective role of AKG against oxidative stress and in supporting follicular development.

**Result:**

AKG treatment advanced puberty onset and increased the number of corpora lutea in mice. It alleviated oxidative stress and apoptosis in granulosa cells by upregulating CAT and downregulating P53. Crucially, OGDH physically interacted with CAT and SOD2 and boosted their enzymatic activities, thereby reinforcing AKG’s antioxidative effects. Knockdown of *OGDH* markedly impaired the ability of AKG to promote follicular development.

**Conclusions:**

These findings identify *OGDH* as a key mediator of AKG’s protective role in follicular development through modulation of oxidative stress and apoptosis. This work provides mechanistic insight into AKG function and supports its potential as a therapeutic strategy for follicular disorders.

**Supplementary Information:**

The online version contains supplementary material available at 10.1186/s40659-026-00688-9.

## Introduction

Follicular disorders reduce the number of follicles [1], cause polycystic ovary syndrome [2] and lead to the decline in female fecundity [3]. Excessive oxidative stress in granulosa cells (GCs) impairs follicular development, affects ovarian reserve, and even leads to follicular disorders [4]. It has been reported that the excess iron in the ovaries increases reactive oxygen species (ROS) levels and induces GC apoptosis, which ultimately leads to follicular development disorders in mice [5]. Conversely, heme oxygenase-1 attenuates heat stress-induced apoptosis in bovine ovarian GCs by decreasing the level of ROS [6], while excessive GC apoptosis diminishes ovarian reserve and precipitates follicular disorders [7]. GCs not only support the development and maturation of follicles [8, 9], but also sustain primordial follicle activation and facilitate follicle formation [10, 11]. Therefore, ROS-induced GC apoptosis is recognized as a key driver of follicular disorders.

α-ketoglutarate (AKG) is an intermediate metabolite of the tricarboxylic acid (TCA) cycle that not only provides energy for cell development but also acts as a potential antioxidant [12, 13]. AKG alleviates oxidative stress and ameliorates osteoarthritis [14], while long-term supplementation preserves ovarian function and delays follicular senescence by reducing ROS and DNA methylation levels [15–17]. Notably, AKG serves as an obligate cofactor for TET dioxygenases, driving DNA demethylation to regulate stem cell fate [18, 19]. Moreover, AKG improves follicle quality and ameliorates follicular disorders by inhibiting pyroptosis of GCs [20], and promotes cardiomyocyte proliferation through JMJD3-dependent demethylation [21]. However, the precise mechanisms underlying AKG’s protective effects on mammalian follicular development remain incompletely understood.

Oxoglutarate dehydrogenase (*OGDH*), a rate-limiting component of the OGDH complex, has been proposed as a mitochondrial redox sensor responsive to ROS [22, 23]. Meanwhile, diabetes reduces the expression of *OGDH* and causes high levels of ROS, thus reducing the proliferation and promoting the apoptosis of cardiac microvascular endothelial cells [24]. It has been reported that OGDH plays a critical role in maintaining metabolic homeostasis and proliferation in PIK3CA-mutant cancer cells [25], and regulates endogenous AKG levels and the apoptosis in MHM cells [26]. Interestingly, low levels of *OGDH* lead to disruption of the TCA cycle and increase the NADP+/NADPH ratio, thereby reducing AKG levels [27, 28]. However, the functional relationship between AKG and *OGDH* in GCs and follicular development disorders is still unclear.

In this study, we investigated the roles of AKG and OGDH in puberty onset and follicular development. We aimed to elucidate how AKG epigenetically regulates OGDH expression via DNA demethylation, and whether OGDH amplifies AKG’s protective effects against oxidative stress-induced follicular disorders. These findings may offer novel therapeutic insights for oxidative stress-related ovarian dysfunction.

## Results

### AKG promoted follicular development in mice

**Fig. 1:**
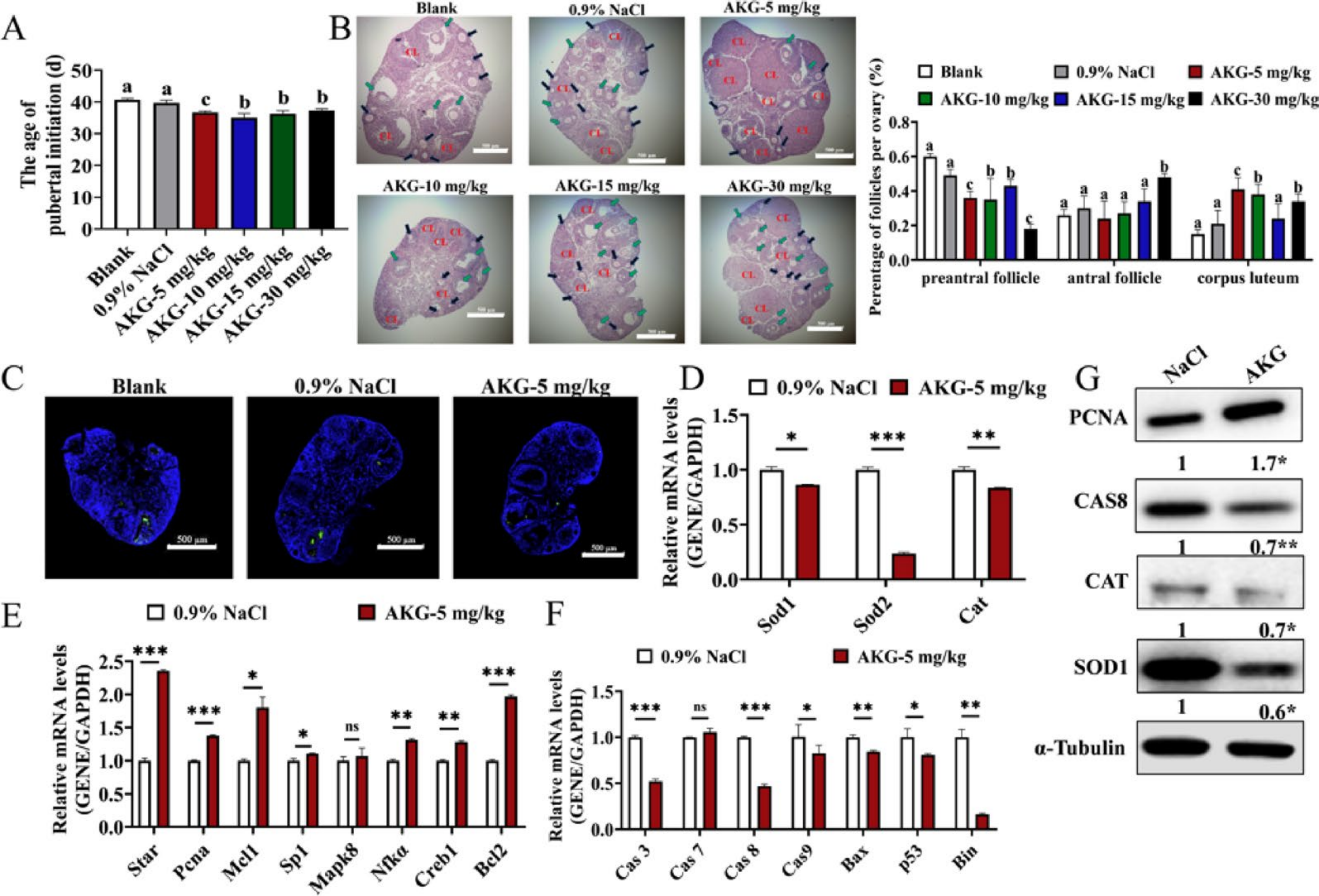
Effects of AKG on mouse follicular development. A. The onset of puberty in mice treated with blank, 0.9 % NaCl, 5 mg/kg, 10 mg/kg, 15 mg/kg, and 30 mg/kg AKG groups (n = 8 mice per group). B. Effects of blank, 0.9 % NaCl, 5, 10, 15, and 30 mg/kg AKG on the development of follicles in mice (scale bar, 500 μm). Left image: photos of mouse ovarian sections stained with hematoxylin and eosin (HE). Black arrows: preantral follicles; Red arrows: antral follicles; CL: corpus luteum. Right image: follicle counts for each stage of development (n = 8 mice per group). C. TUNEL assay of the effect of AKG on the apoptosis of GCs in mouse follicles (scale bar, 500 μm). The mRNA expressions of OS-related (D), proliferation-related (E), and apoptosis-related (F) genes in mouse ovaries. G. The protein expressions of PCNA, CASP8, CAT, and SOD1 in mouse ovaries (n = 3 mice per group). *P < 0.05, **P < 0.01, ***P <0.001, ns, no significant difference.

To investigate the effects of AKG on the development of mammalian ovaries, we administered intraperitoneal injections of AKG at different concentrations (5 mg/kg, 10 mg/kg, 15 mg/kg, 30 mg/kg) to 21-day-old female C57BL/6J mice. Compared with the control group, 5 mg/kg-AKG significantly advanced the onset of puberty in mice (Fig. 1A). Although the numbers of follicles at all developmental stages were decreased, the numbers of corpora lutea were increased by 2.7-fold in mouse ovaries (Fig. 1B). This suggested that 5 mg/kg-AKG was the most effective in promoting follicular development, and was selected for subsequent experiments.

Furthermore, AKG reduced the apoptosis of GCs (Fig. 1C) through inhibiting the mRNA expressions of *Casp3*, *Casp8*, and *Casp9* (Fig. 1F) and the protein expression of CASP8 (Fig. 1G) in mouse ovarian GCs. Meanwhile, it inhibited the mRNA expressions of *Sod1*, *Sod2*, and *Cat* (Fig. 1D) and protein expressions of SOD1 and CAT (Fig. 1G). Conversely, AKG promoted the mRNA expressions of *Star*, *Pcna*, *Mcl1*, *Mapk8*, and *Nfkα* (Fig. 1E) and protein expression of PCNA (Fig. 1G) to promote the proliferation of GCs.

### AKG alleviates oxidative stress and apoptosis and promotes GC proliferation

**Fig. 2:**
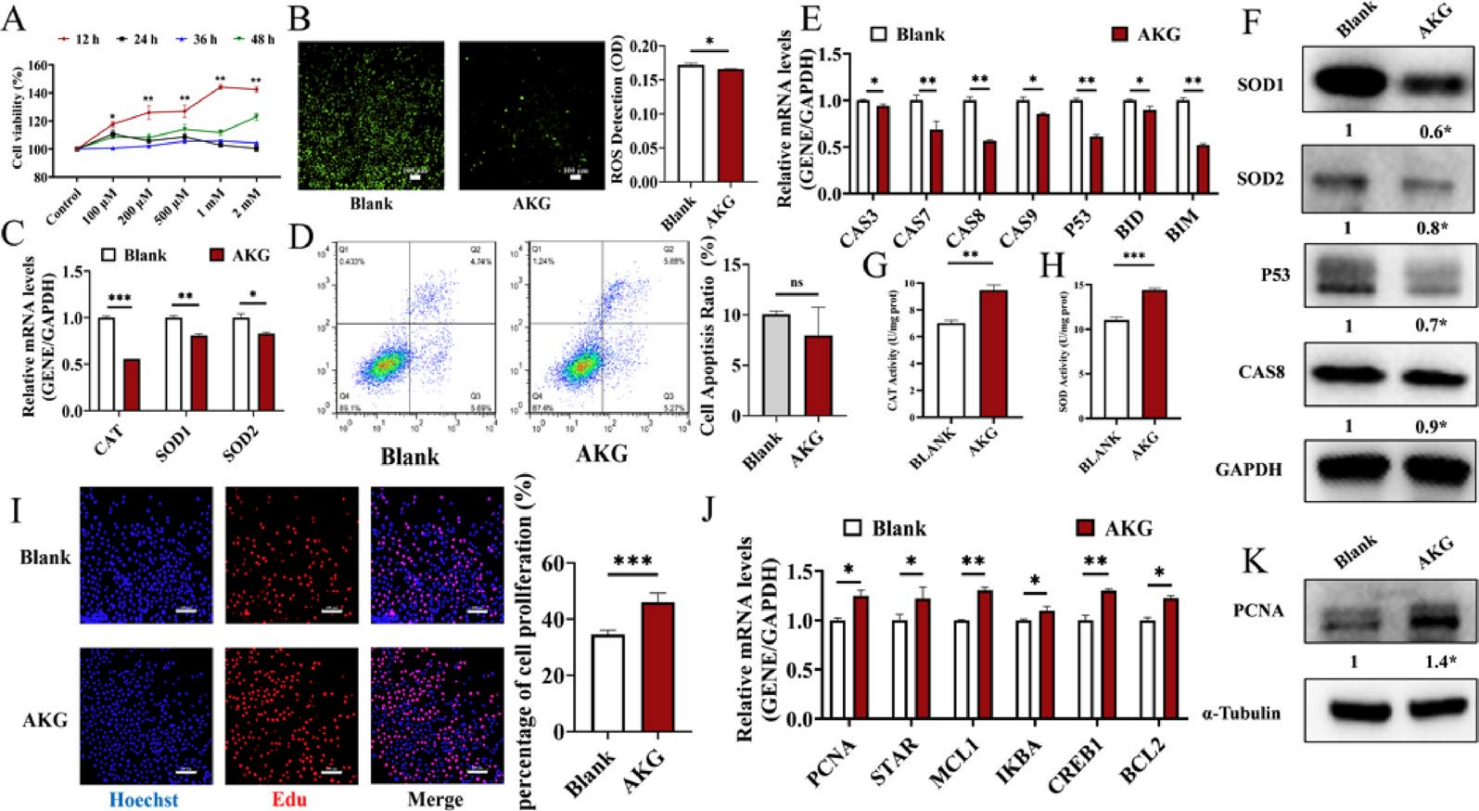
AKG inhibited the apoptosis and OS, and promoted the proliferation in GCs. **A** Cell viability of GCs treated with different concentrations of AKG (AKG; 100, 200, 500 µM, and 1, 2 mM) for 12–48 h. Cell viability was determined using the CCK-8 assay (*n* = 3 biological replicates). **B** The levels of ROS of GCs (*n* = 3, scale bar, 100 μm). **C** The effect of AKG on the mRNA expressions of OS-related genes in GCs (*n* = 3). **D** The apoptosis rates of GCs treated with AKG (*n* = 3). **E** The mRNA levels of apoptosis-related genes in GCs (*n* = 3). **F** The protein expressions of SOD2, SOD1, P53, and CASP8 in GCs. **G** The enzymatic activity of CAT in GCs (*n* = 3). **H** The enzymatic activity of SOD in GCs (*n* = 3). **I** The proliferation rates of GCs assessed by 5-Ethynyl-2′-deoxyuridine (EdU) staining (*n* = 3, scale bar, 100 μm). **J** The mRNA levels of proliferation-related genes (*PCNA*, *STAR*, *MCL1*, and *CREB1*) in GCs (*n* = 3). **K**. The protein expression of PCNA in GCs (*n* = 3). *P < 0.05, **P < 0.01, ***P < 0.001, ns, no significant difference

To further investigate the effects of AKG on the growth of human ovarian GCs, we treated GCs with different concentrations of AKG and found that cell viability was significantly enhanced after exposure to 1 mM AKG for 12 h (Fig. 2A). We then observed that AKG treatment effectively reduced ROS levels in GCs (Fig. 2B). Interestingly, while the mRNA expressions of *SOD1*, *SOD2*, and *CAT* (Fig. 2C) and the protein expressions of SOD1 and SOD2 (Fig. 2F) were significantly downregulated following AKG treatment, we found that AKG significantly enhanced the enzymatic activities of CAT (Fig. 2G) and SOD (Fig. 2H). Meanwhile, AKG inhibited the mRNA expressions of *P53*, *CASP7*, *BIM*, and *CASP8* (Fig. 2E), as well as the protein expressions of P53 and CASP8 (Fig. 2F), leading to a significant decrease in GC apoptosis rates (Fig. 2D). Conversely, AKG improved GC proliferation rates as assessed by EdU staining (Fig. 2I), which was supported by the upregulated mRNA expressions of *PCNA*, *STAR*, *MCL1*, and *CREB1* (Fig. 2J) and the protein expression of PCNA (Fig. 2K).

### AKG improved *OGDH* demethylation and promoted GC proliferation

**Fig. 3:**
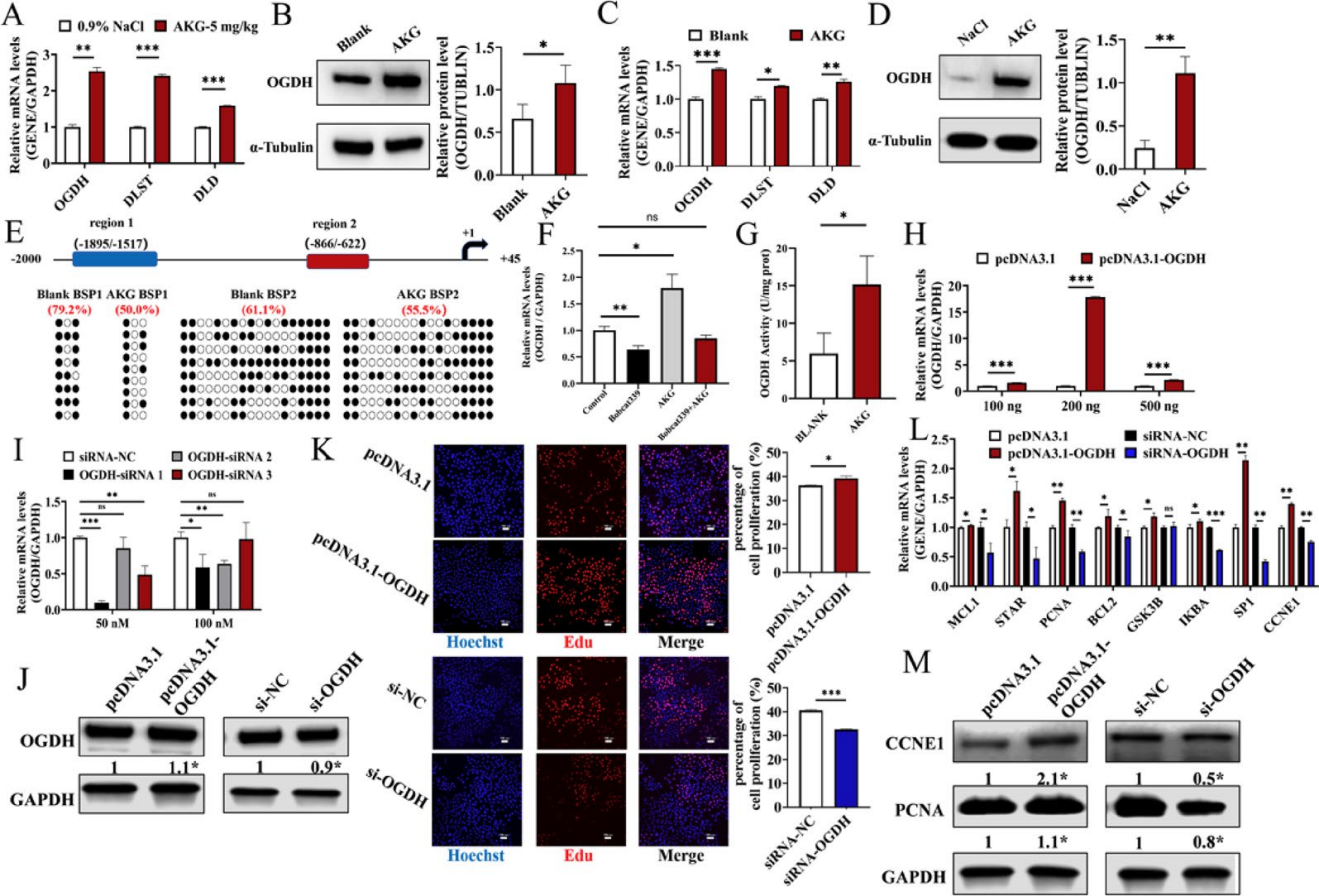
AKG reduced the DNA methylation of *OGDH*. **A**. The mRNA expressions of OGDHC subunits in mouse ovaries treated with AKG (*n* = 3). **B**. The protein expression of OGDH in mouse ovaries treated with AKG. The mRNA **C** and protein **D** expression levels of OGDHC subunits in GCs treated with AKG(*n* = 3).**E**. The methylation status of the *OGDH* promoter. Black circles: methylated; white circles: unmethylated (*n* = 3). **F** The mRNA expression of *OGDH* in GCs treated with AKG in the presence or absence of the TET inhibitor (*n* = 3). **G** The enzymatic activity of OGDH in GCs treated with different concentrations of AKG. The mRNA levels of *OGDH* after overexpression (*n* = 3). **H** and knockdown **I** in GCs. **J**. The protein level of *OGDH* after overexpression and knockdown in GCs (*n* = 3). **K**. The proliferation rates of GCs treated with AKG (*n* = 3, scale bar, 100 μm). **L**. The mRNA expressions of genes in proliferation pathway in GCs (*n* = 3). **M**. The protein expressions of CCNE1 and PCNA in GCs (*n* = 3). *P < 0.05, **P < 0.01, ***P < 0.001, ns, no significant difference

Considering that AKG is a substrate for the OGDHC (2-oxoglutarate dehydrogenase complex), we investigated the effect of AKG on OGDH expression and found that AKG significantly promoted the mRNA and protein expression of OGDH in mouse follicles (Fig. 3A, B) and GCs (Fig. 3C, D). There is a CpG island on the promoter of OGDH, and we found that AKG slightly reduced the methylation level of the OGDH promoter’s CpG island by 5.6% (Fig. 3E). Besides, we treated GCs with AKG in the presence of a specific TET enzyme inhibitor (Bobcat339). As expected, TET inhibition effectively abolished the AKG-induced upregulation of *OGDH* mRNA (Fig. 3F). Consistent with the elevated protein levels, AKG treatment significantly enhanced the catalytic activity of OGDH in GCs (Fig. 3G).

To further reveal the role of *OGDH* in GCs, *OGDH* overexpression vectors (pcDNA3.1-*OGDH*) or *OGDH* small interfering RNAs (*OGDH*-siRNA 1/2/3) were transfected into GCs. The mRNA and protein expression levels of OGDH were significantly increased after transfection with 200 ng pcDNA3.1-OGDH, whereas transfection with 50 nM OGDH-siRNA1 (si-OGDH) significantly reduced OGDH mRNA and protein expression levels (Fig. 3H, I). EdU staining showed that pcDNA3.1-*OGDH* significantly increased the proliferation rates of GCs (Fig. 3K) and upregulated the mRNA expressions of *PCNA*, *SP1*, and *CCNE1* (Fig. 3L) as well as the protein expressions of CCNE1 and PCNA (Fig. 3M). Furthermore, si-*OGDH* reduced the proliferation rates of GCs (Fig. 3K) and inhibited the mRNA expressions of *PCNA*, *IKBA*, *SP1*, and *CCNE1* (Fig. 3L) as well as the protein expressions of CCNE1 and PCNA (Fig. 3M).

### *OGDH* inhibited the apoptosis and oxidative stress of GCs

**Fig. 4:**
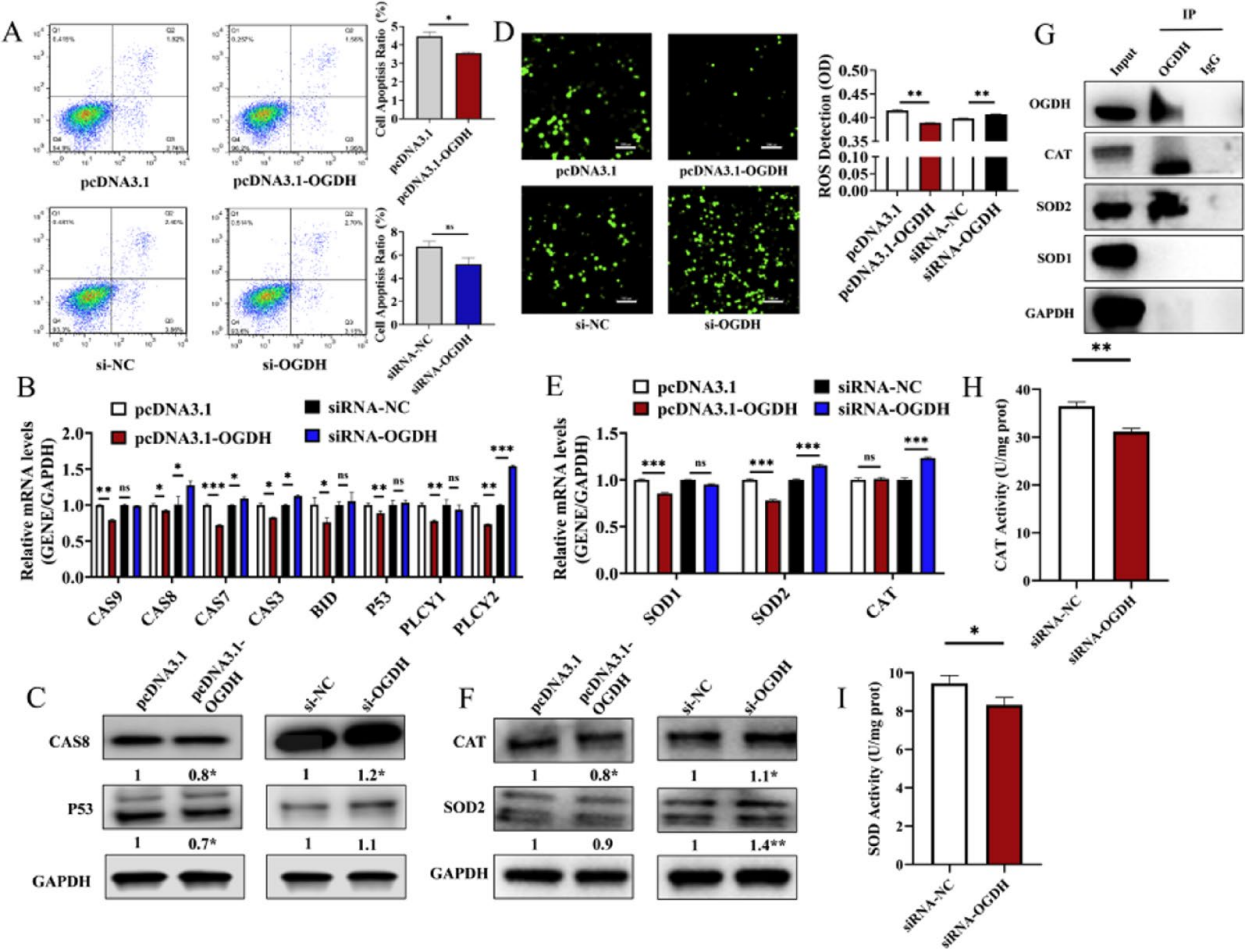
*OGDH* inhibited the apoptosis and oxidative stress of GCs. **A** Flow cytometric analysis of GC apoptosis rates following transfection with OGDH overexpression vector (pcDNA3.1-OGDH) or siRNA (si-OGDH) (*n* = 3). **B**,** C** The mRNA **B** and protein (**C**) expression levels of apoptosis-related markers (*CASP9*,* P53*,* CASP7*,* PLCY1*, and *CASP8*) in GCs (*n* = 3). **D**. The ROS levels of GCs treated with OGDH overexpression vector (pcDNA3.1-OGDH) or siRNA (si-OGDH) (*n* = 3, scale bar, 100 μm). (**E**,**F**) The mRNA **E** and protein **F** expression levels of oxidative stress-related enzymes (*SOD1*,* SOD2*, and *CAT*) (*n* = 3). **G** Co-immunoprecipitation (Co-IP) assay showing the binding between OGDH and antioxidant enzymes (CAT and SOD) (*n* = 3). **H**, **I**. The enzymatic activities of CAT **H** and SOD (**I**) were measured to determine the functional impact of OGDH on antioxidant capacity. (*n* = 3) *P < 0.05, **P < 0.01, ***P < 0.001, ns, no significant difference

We found that pcDNA3.1-OGDH significantly reduced the apoptosis rates of GCs (Fig. 4A) and inhibited the mRNA expressions of *CASP9*, *P53*, *CASP7*, and *PLCY1* (Fig. 4B) as well as the protein expressions of CASP8 and P53 (Fig. 4C), while si-OGDH showed opposite results (Fig. 4B, C). Next, we determined that ROS levels were significantly inhibited when GCs were treated with pcDNA3.1-OGDH (Fig. 4D). Consistent with a potential feedback response to reduced ROS, pcDNA3.1-OGDH decreased the mRNA levels of *SOD1* and *SOD2* (Fig. 4E) as well as the protein expressions of CAT and SOD2 (Fig. 4F). In contrast, si-OGDH increased the expressions of these antioxidant enzymes (Fig. 4E, F), likely as a compensatory response to elevated oxidative stress. Crucially, Co-IP results demonstrated that OGDH co-immunoprecipitated with the CAT and SOD2 proteins (Fig. 4G). Moreover, pcDNA3.1-OGDH significantly enhanced the enzymatic activities of CAT and total SOD, whereas si-OGDH markedly diminished their basal activities (Fig. 4H, I). Together, these data support a functional association between OGDH and the antioxidant enzyme activities of CAT and SOD.

### *OGDH* inhibited the apoptosis and OS, and promoted the proliferation of follicles

**Fig. 5:**
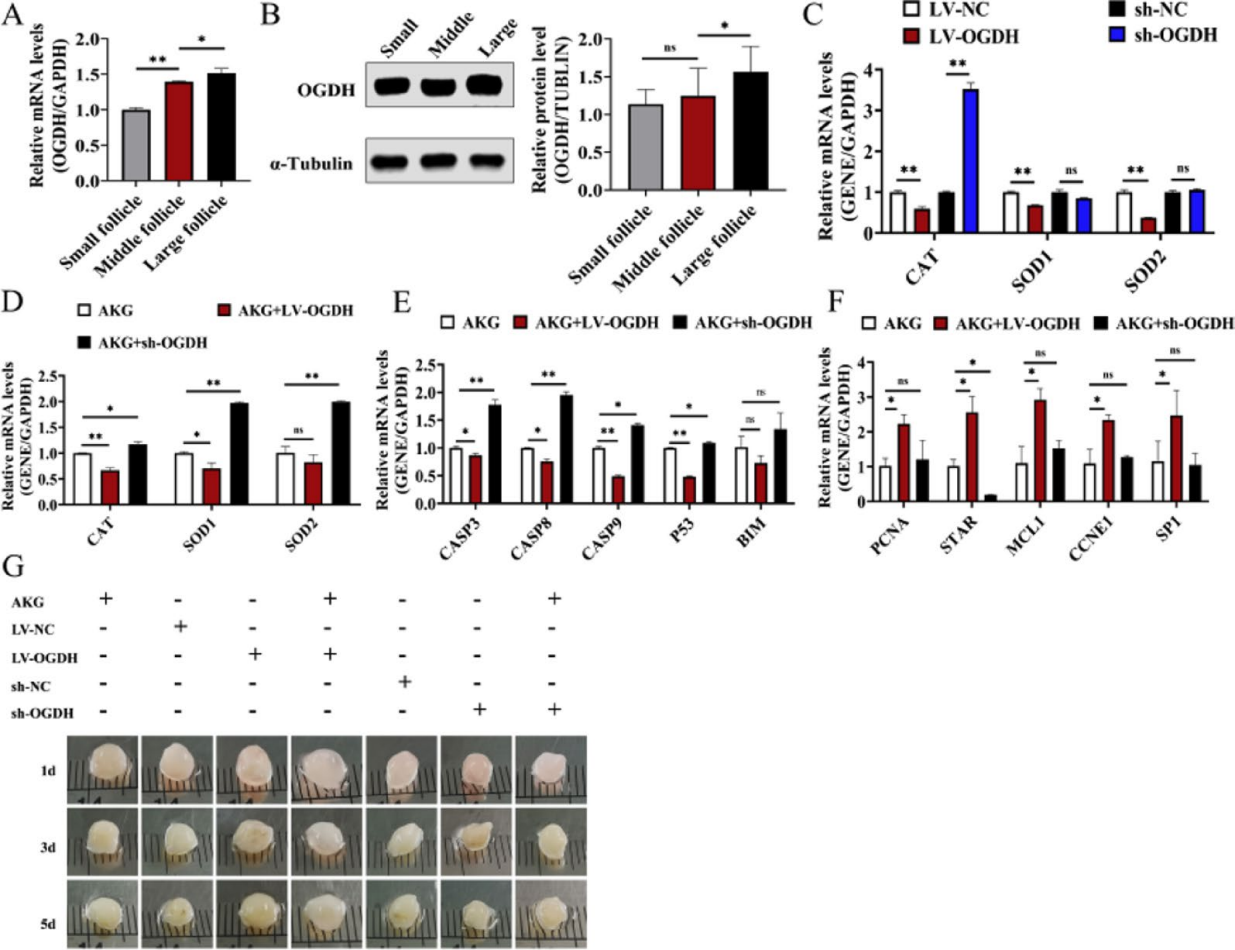
AKG and *OGDH* enhanced the effect of AKG to promote the development of follicles. The mRNA **A** and protein **B** expressions of *OGDH* in small follicles (≤ 3 mm), middle follicles (3–5 mm) and large follicles (≥ 5 mm) (*n* = 3). The expressions of OS-related **C**, **D**, apoptosis-related **E**, and proliferation-related **F** genes in follicles (*n* = 3). **G**. The effects of AKG and *OGDH* lentivirus infection on the development of porcine follicles (*n* = 3). *P < 0.05, **P < 0.01, ***P < 0.001, ns, no significant difference

The mRNA (Fig. 5A) and protein (Fig. 5B) levels of *OGDH* increased gradually across small follicles (1–3 mm), middle follicles (3–5 mm), and large follicles (5–7 mm). Then, the lentiviral vector overexpression of *OGDH* (LV-*OGDH*) and knockdown of *OGDH* (sh-*OGDH*) were constructed and transduced into porcine follicles cultured in vitro. LV-*OGDH* inhibited the mRNA expressions of *CAT*, *SOD1*, and *SOD2*, while sh-*OGDH* showed opposite results (Fig. 5C). Additionally, we observed that LV-*OGDH* enhanced the inhibitory effect of AKG on the mRNA expressions of *CAT*, *SOD1*, and *SOD2*, while sh-*OGDH* impaired the inhibitory effect of AKG on these genes (Fig. 5D). LV-*OGDH* also enhanced the effect of AKG on inhibiting the mRNA expressions of *CASP3*, *CASP8*, *CASP9*, and *P53* (Fig. 5E) and upregulating the mRNA expressions of *PCNA*, *STAR*, *MCL1*, and *SP1* (Fig. 5F). Conversely, sh-*OGDH* disrupted the inhibitory effect of AKG on the expressions of apoptosis-related and proliferation-related genes (Fig. 5E, F).

When follicles were treated with AKG or transduced with *OGDH* lentivirus alone, the follicles remained rounded with few blood vessels. When treated with both AKG and LV-*OGDH*, the follicles maintained a rounded shape, exhibited well-distributed bright red capillaries, and contained clear follicular fluid. However, sh-*OGDH* compromised the positive effect of AKG in promoting follicular development and decreasing the loss of follicular blood vessels (Fig. 5G).

### *OGDH* promoted the onset of puberty and the development of follicles in mice

**Fig. 6:**
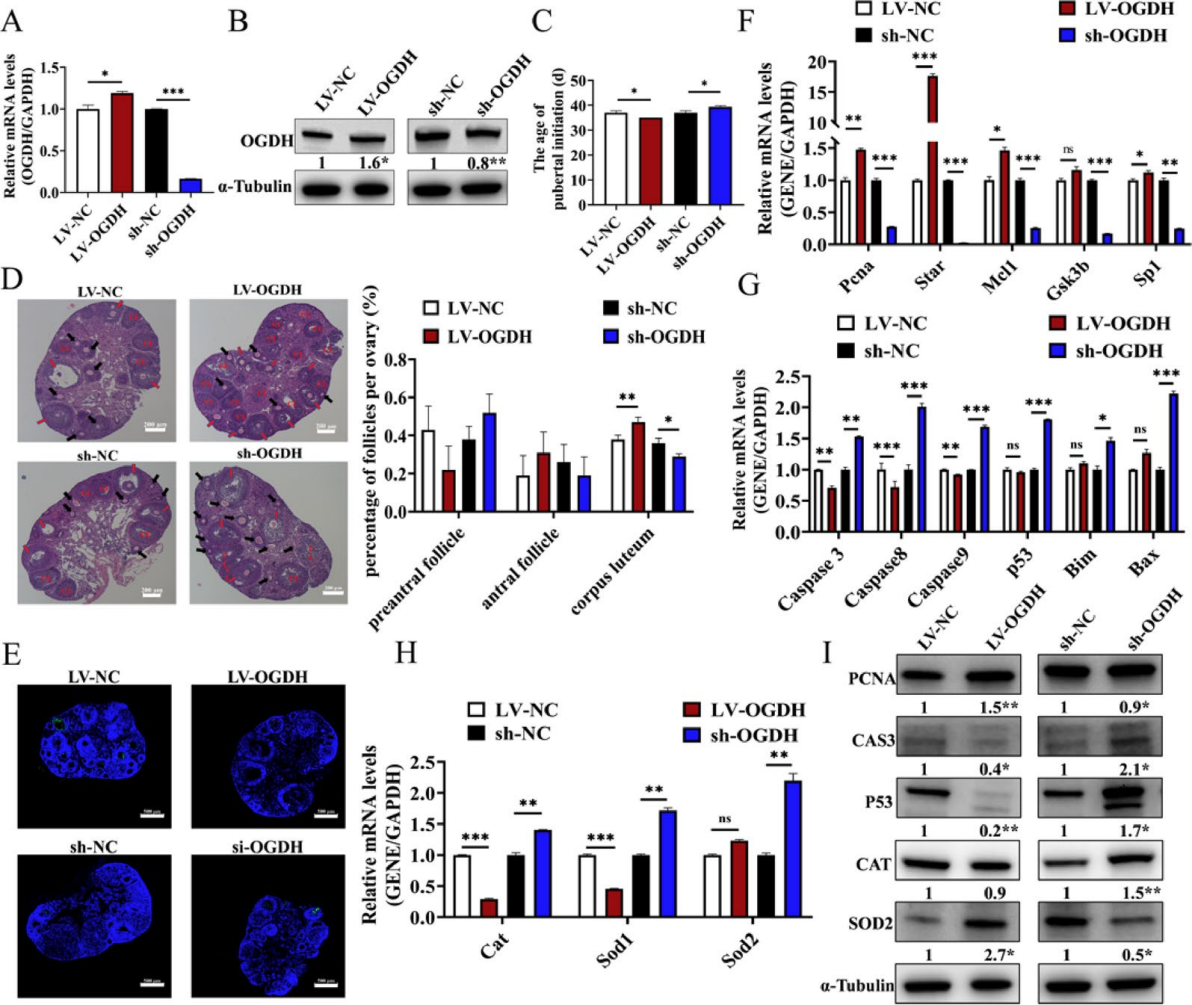
*OGDH* promoted follicular development in mice. The mRNA **A** and protein **B** expressions of *OGDH* in mouse follicles (*n* = 3). **C**. The onset of puberty after ovarian transfection with LV-*OGDH* and sh-*OGDH* in mice (*n* = 8 mice per group). **D.** Left: mouse ovarian sections stained with HE (*n* = 3, scale bar, 200 μm). Black arrows: preantral follicles; Red arrows: antral follicles; CL: corpus luteum. Right: follicle counts for each stage of development (*n* = 8 mice per group). **E**. The apoptosis of GCs transfection with LV-*OGDH* and sh-*OGDH* in mice (*n* = 3, scale bar, 500 μm). The mRNA expressions of proliferation-related **F**, apoptosis-related **G**, and OS-related **H** genes in GCs (*n* = 3). **I**. The protein expressions of proliferation-related, apoptosis-related, and OS-related genes in GCs transfection with LV-*OGDH* and sh-*OGDH* in mice (*n* = 3). *P < 0.05, **P < 0.01, ***P < 0.001, ns, no significant difference

To further verify the role of *OGDH* on follicular development, the LV-*OGDH* and sh-*OGDH* lentiviral vectors were injected into mice via intraperitoneal injection. The mRNA (Fig. 6A) and protein (Fig. 6B) expressions of *OGDH* in the LV-*OGDH* group were significantly higher than those in the LV-NC group, while sh-*OGDH* significantly reduced the mRNA (Fig. 6A) and protein (Fig. 6B) expressions of OGDH compared with the sh-NC group indicating that LV-*OGDH* and sh-*OGDH* had been successfully transduced into mouse follicles.

We found that LV-*OGDH* advanced the onset of puberty and significantly increased the number of corpus luteum, while sh-*OGDH* delayed the onset of puberty and decreased the number of corpus luteum in mouse follicles (Fig. 6C, D). LV-*OGDH* also reduced the apoptosis of GCs (Fig. 6E) and decreased the mRNA expressions of *Casp3*, *Casp8*, *Casp9*, and *p53* (Fig. 6G) as well as the protein expressions of CASP3 and P53 (Fig. 6I). Moreover, LV-*OGDH* increased the mRNA expressions of *Pcna*, *Star*, *Mcl1*, and *Sp1* (Fig. 6F) as well as the protein expression of PCNA (Fig. 6I) to enhance the proliferation of GCs, while LV-*OGDH* inhibited the mRNA expressions of *Sod1* and *Cat* (Fig. 6H) as well as the protein expressions of CAT and SOD1 (Fig. 6I) to reduce the oxidative stress of GCs in mouse follicles. In contrast, sh-*OGDH* led to the opposite results (Fig. 6E, H, I).

## Discussion

Accumulating studies have shown that paracrine factors released by GCs stimulate primordial follicle formation and support follicular development [29]. AKG has been shown to reduce ROS levels and cell apoptosis to ameliorate cardiac systolic disorders [30] and improve abdominal aortic aneurysm in mice [31]. AKG also promotes cell growth and rescues abnormal folliculogenesis [32]. In our study, we found that intraperitoneal administration of 5 mg/kg-AKG significantly advanced the onset of puberty (Fig. 1A) and promoted a 2.7-fold increase in the number of corpora lutea (Fig. 1B), compared with the control group. Regarding the dosing strategy, while dietary supplementation is common, we selected intraperitoneal injection to ensure precise bioavailability. Recent studies indicate that intraperitoneal administration of AKG achieves circulating levels comparable to those obtained through dietary supplementation [33], and no systemic toxicity was observed at our selected dosage, supporting the safety and physiological relevance of this approach.

Studies have shown that oxidative stress increases the proportion of antral follicles and the number of apoptotic GCs, ultimately causing follicular disorders [33, 34]. Drawing on previous findings that identified OGDH as a “mitochondrial redox sensor” [23], we investigated whether AKG modulates the intracellular redox state through OGDH. Our results demonstrated that AKG upregulated *OGDH* expression via promoter demethylation (Fig. 3E) and significantly enhanced the enzymatic activity of OGDH (Fig. 3G). Crucially, OGDH was found to co-immunoprecipitate with CAT and SOD2 (Fig. 4G), and knockdown of OGDH diminished the enzymatic activities of CAT and SOD (Fig. 4H, I). Although NAD(H)/NADP(H) redox couples, and metabolic flux were not performed in this study, our results provide functional evidence supporting OGDH-mediated enhancement of CAT and SOD2 activity by which AKG alleviates oxidative stress in GCs.

Interestingly, this enhancement in enzymatic activity was accompanied by a reduction in SOD1 and CAT protein levels (Fig. 2F). Consistent with this observation, similar reductions in antioxidant enzyme transcript levels have been reported with other antioxidants such as kynurenic acid (KYNA), which reduced SOD2 and CAT transcript levels following ROS mitigation [35]. Concurrently, AKG enhanced PCNA expression (Figs. 1E and G and 2J) to promote GC proliferation (Fig. 2I). Given that oxidative stress is a primary driver of ovarian senescence [17], these findings are consistent with the broader literature identifying AKG as a potent anti-aging metabolite [12]. Furthermore, unlike NAD⁺ boosters which exert protective effects primarily via sirtuin activation [36], AKG protects GCs by directly enhancing antioxidant enzyme efficiency through OGDH, thereby re-establishing the balance between oxidative stress and cell survival.

Mechanistically, to understand how AKG protects against this GC apoptosis, we examined the upstream regulation of *OGDH*. It is well established that AKG serves as an obligate cofactor for TET enzymes to actively drive DNA demethylation [19] Interestingly, while other TCA cycle intermediates, such as succinate and fumarate, act as competitive inhibitors of TET dioxygenases to promote DNA hypermethylation and cellular dysfunction [37], AKG specifically promoted OGDH transcription by reducing the methylation level of the CpG island within the OGDH promoter by 5.6% (Fig. 3E). To verify this epigenetic regulation, we utilized a specific TET inhibitor, which effectively abolished the AKG-induced upregulation of *OGDH* (Fig. 3F). We note that 5-hydroxymethylcytosine (5-hmC) levels and ChIP-based analyses of promoter occupancy or histone marks were not directly assessed in the current study, which warrants further investigation to fully map this epigenetic landscape.

While the mouse model allowed for the assessment of systemic phenotypes, the porcine model was specifically selected for follicle culture due to its high anatomical and physiological similarity to human ovarian dynamics. Importantly, by utilizing porcine follicles obtained from local abattoirs and employing human cell lines, our multi-model approach significantly reduces the reliance on in vivo mouse models. This minimizes animal sacrifice, strictly complying with the 3R ethical principles. Crucially, the amino acid sequence of OGDH is highly conserved (> 90%) across mouse, pig, and human. While we acknowledge potential species-specific differences, this molecular conservation robustly supports our conclusion that the AKG-mediated OGDH represents an evolutionarily conserved mechanism in mammalian follicular development.

We found that LV-OGDH mirrored the systemic benefits of AKG, advancing the onset of puberty and the number of corpora lutea by 24% in mouse ovaries (Fig. 6C). At the cellular level, *OGDH* overexpression reduced ROS levels and promoted proliferation (Fig. 3I, K). These findings strongly align with previous studies in oncology, where *OGDH* knockdown was shown to increase ROS and inhibit cancer cell viability [25], whereas its upregulation promoted cell proliferation and migration [26]. In our study, we found that LV-*OGDH* enhanced the effect of AKG in alleviating oxidative stress and apoptosis by reducing the expressions of CAT (Fig. 5D) and CASP9 (Fig. 5E), and enhanced the promotive effect of AKG to promote proliferation by increasing the expressions of *PCNA* and *STAR* in follicles (Fig. 5F). In contrast, sh-OGDH abolished the protective effects of AKG. These data suggested that OGDH is the critical effector enhancing the activity of AKG to inhibit oxidative stress and apoptosis while promoting the proliferation of GCs.

We acknowledge certain limitations in our current experimental framework. We captured global oxidative stress in ROS analysis but did not use mitochondrial superoxide indicators like MitoSOX, which can be access in the future. Secondly, given the structural similarity of OGDH to OGDHL and DHTKD1, the potential redundant or synergistic roles of these isoenzymes warrant further investigation. Additionally, from a statistical perspective, conducting formal assessments of data normality and homogeneity of variance prior to hypothesis testing would further enhance the statistical rigor. Ultimately, despite these limitations, our evidence establishes that AKG epigenetically upregulates *OGDH* transcription via DNA demethylation, thereby reducing oxidative stress and promoting GC survival and proliferation to protect follicular development.

## Conclusion

Overall, both AKG supplementation and OGDH overexpression accelerated puberty onset and ameliorated follicular disorders by reducing ROS levels, inhibiting apoptosis, and promoting proliferation in GCs and follicles. Mechanistically, AKG induced the demethylation of the *OGDH* promoter, thereby upregulating *OGDH* expression. These findings might provide useful information to promote follicular development and relieve follicular disorders.

## Materials and methods

### Animal experiment

#### AKG interference mouse experiment

Three-week-old female C57BL/6J mice (*n* = 48) were obtained from Guangdong YaoKang Biotechnology (China) and randomly divided into six groups (*n* = 8 per group): blank control, vehicle control (0.9% NaCl), and AKG treatment groups at doses of 5, 10, 15, or 30 mg/kg. AKG (Sigma-Aldrich, USA) was dissolved in 0.9% NaCl. The dosage range of 5–30 mg/kg via intraperitoneal injection was selected based on pharmacokinetic evidence that a 10 mg/kg intraperitoneal injection dose yields circulating AKG concentrations comparable to chronic 2% dietary supplementation [38], thereby ensuring physiological relevance without causing systemic toxicity. Mice were administered AKG or vehicle via intraperitoneal injection at a volume of 0.1 mL, three times per week for 3 weeks. Pubertal initiation was determined by vaginal opening.

#### *OGDH* lentiviral interference mouse experiment.

Three-week-old female C57BL/6J mice (*n* = 32) obtained from Guangdong YaoKang (China) and randomly divided them into LV-NC (*n* = 8), LV-OGDH (*n* = 8), sh-OGDH (*n* = 8), and sh- NC (*n* = 8) groups. The lentiviral vector that overexpression or knockdown of OGDH (LV-OGDH or sh-OGDH) was synthesized by Dongze Biotechnology Co., LTD (Guangzhou, China). The lentivirus was injected in the same way as our previous study [39]. Briefly, 1 × 10^7^ TU of lentivirus was infected into mice via intraperitoneal injection and was given once a week for 3 weeks. Similar to the AKG experiment, body weight and general health were monitored throughout the study. In this study, the pubertal initiation of mice was characterized by the opening of vaginal orifice.

### Isolation and culture of GCs and follicles

Collect healthy ovaries from local abattoirs and wash them twice with PBS (Invitrogen, Shanghai, China) containing 1% streptomycin/penicillin, and transported to the cell chamber. The cells cultured in this study were human ovarian granulosa cells, called GCs in this study, were purchased from Wuhan Punose Life Science Technology. The cells were cultured with Dulbecco’s Modified Eagle’s Medium (DMEM, Hyclone, Logan, UT, USA) containing 10% fetal bovine serum (FBS, Hyclone, Logan, UT, USA) and 1% penicillin-streptomycin (Hyclone, Logan, UT, USA). The GCs were incubated at 37 ℃ under 5% CO_2_. The cell state was observed, and when the confluency reached 80%, plasmids were transfected into GCs through LipofectamineTM 3000 (Thermo Scientific, USA). The 3–5 mm antral follicles separated from ovaries were cultured in 24-well plates with serum-free DMEM/F12 without fetal bovine serum (FBS) at 37 °C under 5% CO_2_. The follicle status and photos were observed at days 1, 3, and 5 after lentivirus infection or drug treatment.

### Hematoxylin-eosin staining and TUNEL assay

Each mouse ovary was fixed with 4% paraformaldehyde for 24 h. They were then embedded with 5 μm thick paraffin sections and stained with hematoxylin and eosin staining (H&E), and the morphological characteristics of the ovary were observed under microscope.

The TUNEL apoptosis detection assay was performed according to manufacturer’s instructions in the TUNEL Apoptosis Detection Kit (Beyotime, China). The ovarian cysts in the paraffin-embedded mouse ovarian block were cut into paraffin-embedded sections, and separately incubated with xylene for 10 min, ethanol for 5–10 min, and proteinase K for 15 min. After washing, the sections were incubated with the TUNEL reagent in the dark for 1 h. After washing with PBS, the nuclei were counterstained with DAPI for 5 mintues. Finally, the sections were placed under an Nikon ECLIPSE Ti2 fluorescence microscope for imaging.

### EdU assay

Granulosa cell (GC) proliferation was assessed using a Cell-Light™ EdU Apollo Kit (RiboBio, China) according to the manufacturer’s protocol. GCs were seeded into 48-well plates and incubated under standard culture conditions. Cells were then incubated with EdU working solution for 30 min, fixed with 4% paraformaldehyde for 15 min, and permeabilized with 0.5% Triton X-100 for 10–15 min at room temperature. After washing with PBS, the cells were stained with Apollo reaction buffer for 30 min and counterstained with Hoechst for 10 min. Images were captured using a fluorescence microscope. The percentage of EdU-positive nuclei relative to total Hoechst-stained nuclei was calculated from three randomly selected fields per well to determine the proliferation rate.

### Flow cytometry assay

The apoptosis rate of GCs was detected by Annexin V-FITC Apoptosis Detection Kit (BioVision, Milpitas, CA, USA). Cells were collected and washed twice with PBS. The cells were re-suspended with 500 µL 1×Annexin V buffer. cells were added with 5 µL Annexin V-FITC and 5 µL PI staining solution and incubated in darkness for 15 min. The apoptosis of cells was detected and analyzed by flow cytometry ((guava easyCyte, Luminex, USA), and the total number of early and late apoptosis was calculated.

### Reactive oxygen species assay

Intracellular reactive oxygen species (ROS) levels were measured using the Active Oxygen (ROS) Detection Kit (Beyotime, China) following the manufacturer’s instructions with minor modifications. Briefly, cells were seeded in culture plates and, after adherence, were transfected or treated with the indicated drugs for 24 h. The probe was diluted 1:1000 in serum-free culture medium to prepare the working solution. After removing the culture medium, 100 µL of working solution was added to each well, and cells were incubated for 20 min at 37 °C in the dark. After incubation, the probe solution was aspirated, and cells were gently washed three times with serum-free culture medium to remove excess extracellular dye. Fluorescence signals were then detected at an excitation wavelength of 488 nm using a fluorescence microplate reader, and the fluorescence intensity was recorded for quantitative analysis. In parallel, cells were visualized under a fluorescence microscope and representative images were captured under identical exposure settings across groups. Background signals from dye-only blanks were subtracted, and data were collected from at least three independent experiments.

### Cell viability assay

The viability of GCs was determined using the Cell Counting Kit-8 (CCK-8; Biosharp, China). Cells were seeded into 96-well plates at a density of 5 × 10³ cells/well and treated with various concentrations of α-ketoglutarate (100 µM, 200 µM, 500 µM, 1 mM, and 2 mM) for 12, 24, 36, or 48 h. After incubation, 10 µL of CCK-8 reagent was added to each well and incubated for 2 h at 37 °C. The absorbance at 450 nm was measured using a microplate reader (Bio-Rad, USA), and cell viability was expressed as a percentage relative to the control group.

### Co-Immunoprecipitation

Co-immunoprecipitation was performed using a Co-IP Kit (Bioss Biotechnology, China) following the manufacturer’s instructions. GCs were transfected with plasmids using X-tremeGENE™ transfection reagent (Roche, 06366236001) for 24 h. Cells were lysed on ice in IP lysis buffer supplemented with protease inhibitors (Beyotime, China). The lysates were pre-cleared with Protein A agarose beads for 2 h at 4 °C and then incubated overnight at 4 °C with 2 µg of anti-OGDH (Proteintech, China) as the bait or normal rabbit IgG as a negative control. The immune complexes captured by the OGDH antibody, consisting of OGDH and its bound proteins, were subsequently precipitated using Protein A agarose beads for 2 h at 4 °C with gentle rotation. Beads were washed once with lysis buffer and three times with wash buffer, and bound proteins were eluted by boiling in SDS loading buffer. The precipitated proteins were analyzed by Western blot using antibodies against CAT and SOD2 to verify the binding of OGDH with target proteins.

### Bisulfite sequencing PCR (BSP)

Bisulfite conversion was performed on genomic DNA from granulosa cells (GCs) using the EZ DNA Methylation-Gold™ Kit (Zymo Research, USA) according to the manufacturer’s protocol. Approximately 1 µg of gDNA was converted at 64 °C for 2.5 h and eluted per kit instructions. The bisulfite-converted DNA was then used as template to amplify two OGDH promoter regions with BSP primers (Table 1). PCR cycling was 95 °C for 3 min; 35 cycles of 95 °C for 30 s, 54.9 °C (region 1) or 52.7 °C (region 2) for 30 s, and 72 °C for 30 s; followed by 72 °C for 5 min. Amplicons were purified and ligated into pMD18-T (Takara) using 1 µL vector, 4 µL insert, and 5 µL Solution I (mix DNA and vector thoroughly before adding Solution I), and the ligation was incubated at 16 °C for 5 h. The ligation mixture was then transformed into Trans5α chemically competent E. coli and colony PCR was performed to identify positive clones The confirmed colonies were subjected to Sanger sequencing followed by sequence alignment/analysis. CpG methylation was quantified with QUMA (http://quma.cdb.riken.jp/); for each clone, the methylation ratio was calculated as methylated CpGs / total CpGs. BSP primer sequences are listed in Table 1.

**Table 1:** Primers used for BSP

Gene name	Primer sequences (5′ to 3′)	Size (bp)
Region 1	F: GTTAGGTATGGTGGAGCGTGTTAG	343 bp
	R: CCCTTCTCCTCCTATCCTTCTCTC	
Region 2	F: GTTGGAGTGTAATGGTACGATTTCG	245 bp
	R: AACCGAACGCGATAACTCACG	

### Western blot analysis

Total protein concentration was determined using a BCA Protein Assay Kit (Beyotime, China). Equal amounts of protein from each sample were separated by SDS–PAGE and electrophoresed at 130 V for 45 min, then transferred onto PVDF membranes using a wet transfer system. Prior to transfer, PVDF membranes were activated in methanol for 5 min and equilibrated in transfer buffer. After transfer, membranes were blocked with 5% (w/v) skim milk in TBST at 37 °C for 1 h, washed three times with TBST (5 min each), and incubated with primary antibodies diluted in TBST at 4 °C overnight. Membranes were then washed and incubated with HRP-conjugated secondary antibodies (goat anti-mouse IgG, ab6789, Abcam, 1:500; or goat anti-rabbit IgG, ab205718, Abcam, 1:10,000) at 37 °C for 2 h.

Protein bands were visualized using a Tanon 5200 Multi imaging system (Shanghai, China) and quantified by densitometry using ImageJ. Target protein signals were normalized to the corresponding internal loading control (GAPDH or β-actin) in the same lane to correct for loading variations. Relative expression levels were calculated using a ratio-of-ratios approach.

$$ {\mathrm{Relative}}\:{\text{expression = }}\frac{{\left( {{\mathrm{I}}_{{{\mathrm{target}}}} {\mathrm{/I}}_{{{\mathrm{LC}}}} } \right)}}{{\left( {{\mathrm{I}}_{{{\mathrm{target}}}} {\mathrm{/I}}_{{{\mathrm{LC}}}} } \right)_{{{\mathrm{Control}}}} }} $$  

where ${I}_{\mathrm{target}}$is the band intensity of the target protein, ${I}_{\mathrm{LC}}$is the band intensity of the loading control (GAPDH or β-actin), and the Control group mean was set to 1.

### Enzyme activity assay

Cells were treated with either α-ketoglutarate (α-KG, 1 mM) for 12 h or the TET inhibitor Bobcat339 (5 µM) for 24 h. After treatment, cells were harvested, washed with ice-cold PBS, and collected at 1 × 10^6 cells per sample. Cells were resuspended in ice-cold extraction buffer supplied with the assay kit and lysed by sonication on ice. The lysates were centrifuged at 11,000 × g for 10 min at 4 °C, and the supernatants were collected immediately for subsequent enzyme activity measurement.

The CAT and OGDH activity were measured by using commercial kits from Solarbio Science & Technology (Beijing, China) and the total SOD activity was measured using a commercial kit (Elabscience, E-BC-K020-M) according to the manufacturer’s instructions, respectively. Assays were performed in UV-transparent 96-well plates, and absorbance was measured using a microplate reader (Synergy H1, supplied by Gene Company Limited).

### Quantitative reverse transcription PCR

Total RNA from cells and tissues was extracted and collected with TRIzol reagent (TaKaRa, Tokyo, Japan). With the RevertAid First Strand cDNA Synthesis Kit (Thermo Scientific, Waltham, MA, USA), the extracted RNA was reverse-transcribed into cDNA. The Maxima SYBR Green qRT-PCR master mixture (2×) (Thermo Science) was used to quantify the relative expression levels of mRNA in a photocyclic real-time PCR system. Using GAPDH as endogenous control, 2^−ΔΔct^ strategy was applied for the analysis of expression levels. The primers are shown in Table 2.

**Table 2:** Primers used for qPCR

Gene name	Primer sequences (5′ to 3′)	Size (bp)
*OGDH*	F: GGGGACACCGAGGGGAAGAAG	133 bp
	R: CGTGGACAGTGCCGTGAGTTG	
*CAT*	F: AACTGTCCCTTCCGTGCTA	202 bp
	R: CCTGGGTGACATTATCTTCG	
*SOD1*	F: ACCTGGGCAATGTGACTG	176 bp
	R: TCCAGCATTTCCCGTCT	
*SOD2*	F: GGACAAATCTGAGCCCTAACG	159 bp
	R: CCTTGTTGAAACCGAGCC	
*GAPDH*	F: GGACTCATGACCACGGTCCAT	220 bp
	R: TCAGATCCACAACCGACACGT	
*PCNA*	F: GCAGAGCATGGACTCGTCTC	120 bp
	R: TTGGACATGCTGGTGAGGTT	
*BCL-2*	F: TTGCCGAGATGTCCAGCCAG	202 bp
	R: TCAGTCATCCACAGGGCGAT	
*MCL1*	F: GAAGGCGTTAGAGACCCTGC	167 bp
	R: TGCCCCAGTTTGTTACTCCG	
*CCNE1*	F: ACTGATGTCTCTGTTCGCTCC	175 bp
	R: TGTCAGGTGTGGGAATGAAGG	
*STAR*	F: AGACTTTGTGAGTGTGCGCT	254 bp
	R: AGTCCACCTGGGTCTGTGAT	
*Casp3*	F: GGATTGAGACGGACAGTGGG	124 bp
	R: CCGTCCTTTGAATTTCGCCA	
*Casp8*	F: CTCTGCCTACAGGGTCATGC	162 bp
	R: AGGATGGCCCTCTTCTCCAT	
*Casp9*	F: AACTTCTGCCATGAGTCGGG	128 bp
	R: CTGGCCTTGGCAGTCAGG	
*P53*	F: ACGCTTCGAGATGTTCCGAG	137 bp
	R: TTTTATGGCGGGAGGGAGAC	
*Bax*	F: AGCGCATTGGAGATGAACTG	157 bp
	R: AAGTAGAAAAGCGCGACCAC	

#### Statistical analysis

Statistical analyses were performed using GraphPad Prism 9.0 (GraphPad Software, USA). Data are presented as mean ± standard deviation (SD). For comparisons among more than two groups, One-way Analysis of Variance (ANOVA) was performed. For comparisons between two independent groups, a two-tailed Student’s t-test was used. All experiments were performed with a minimum of three biological replicates (*n* ≥ 3). * indicated a significant difference between the two groups, *P* < 0.05. ** indicated a highly significant difference between the two groups, *P* < 0.01. *** indicated a very significant difference between the two groups, *P* < 0.001.

## Supplementary Information

Below is the link to the electronic supplementary material.

Supplementary Material 1

Supplementary Material 2

Supplementary Material 3

Supplementary Material 4

Supplementary Material 5

Supplementary Material 6

## Data Availability

As requested, the authors will make all data and materials available to other researchers.
