# Supplementary material for: OGDH mediates α-ketoglutarate-induced follicular development and antioxidative response by interacting with CAT/SOD2
Source: Biol Res. 2026 Apr 10;59:33. doi: 10.1186/s40659-026-00688-9 (PMC13200353; doi:10.1186/s40659-026-00688-9)

1. PCNA (Treated with NaCl/AKG)





1. CASP8 (left side, treated with NaCl/AKG)





3.CAT (right side, treated with NaCl/AKG)





4.SOD1(treated with NaCl/AKG)





5.TUBULIN


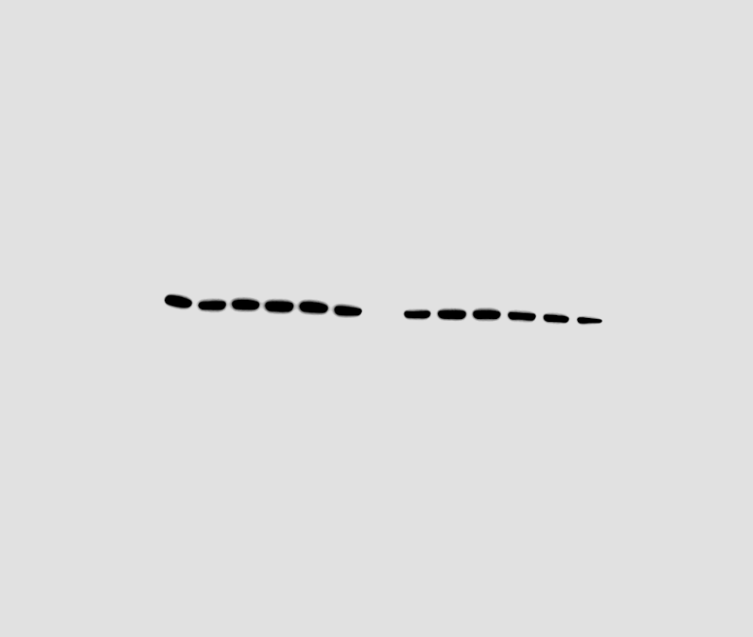

Supplement: Supplementary file 1 — Supplementary Material 1 [file 40659_2026_688_MOESM1_ESM.docx]
