# Supplementary material for: OGDH mediates α-ketoglutarate-induced follicular development and antioxidative response by interacting with CAT/SOD2
Source: Biol Res. 2026 Apr 10;59:33. doi: 10.1186/s40659-026-00688-9 (PMC13200353; doi:10.1186/s40659-026-00688-9)
Supplement: Supplementary file 2 — Supplementary Material 2 [file 40659_2026_688_MOESM2_ESM.docx]

1.SOD1 (Blank/AKG)





2.SOD2 (Blank/AKG)





3.P53(right side，Blank/AKG)





4.CASP8(Blank/AKG)





5.GAPDH





6.PCNA (Blank/AKG)





7.Tubulin
