# Supplementary material for: OGDH mediates α-ketoglutarate-induced follicular development and antioxidative response by interacting with CAT/SOD2
Source: Biol Res. 2026 Apr 10;59:33. doi: 10.1186/s40659-026-00688-9 (PMC13200353; doi:10.1186/s40659-026-00688-9)

3B

1.OGDH（Blank/AKG）





2.TUBULIN





3D

1.OGDH (NaCl/ AKG)





2.TUBULIN


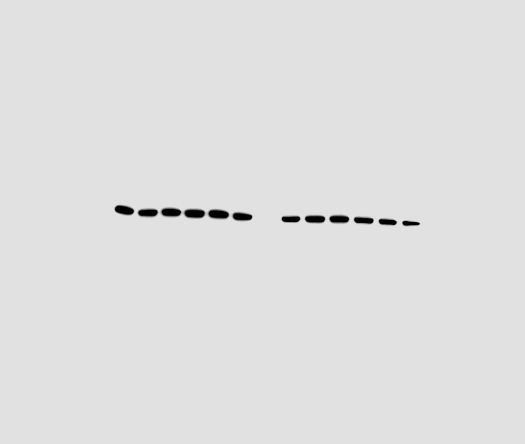


3H

1.OGDH(left side pcDNA3.1/pcDNA3.1-*OGDH*，right side si-NC/si-*OGDH*)


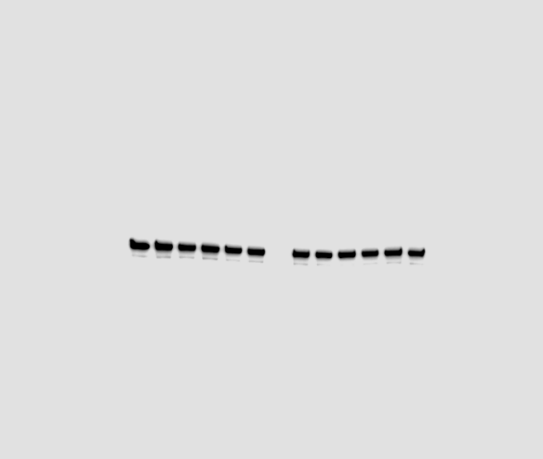


2.GAPDH


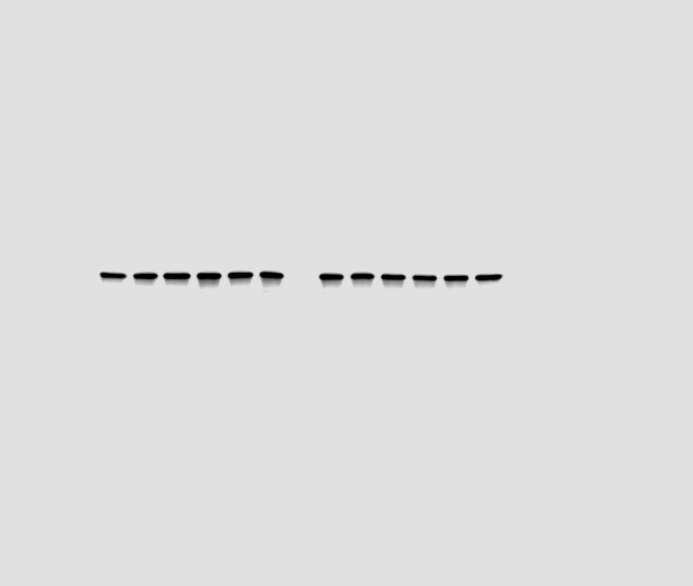


3K

1.CCNE1(upper bands，left side pcDNA3.1/pcDNA3.1-*OGDH*,right side si-NC/si-*OGDH*)


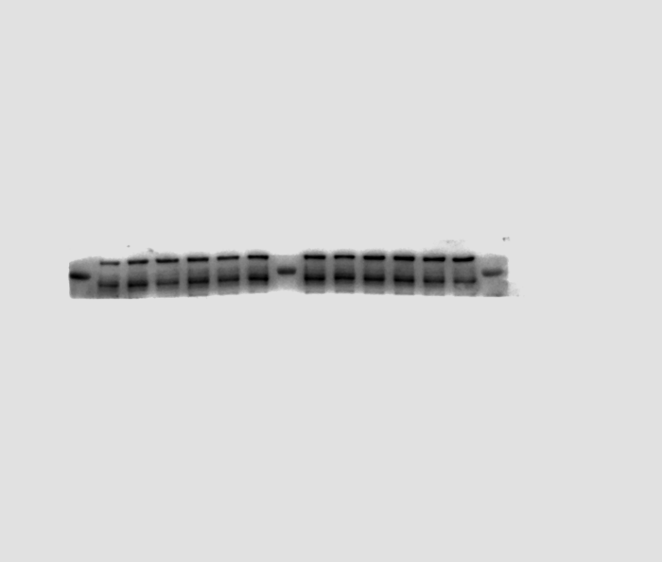


2.PCNA (left side pcDNA3.1/pcDNA3.1-*PCNA*,right side si-NC/si-*PCNA*)


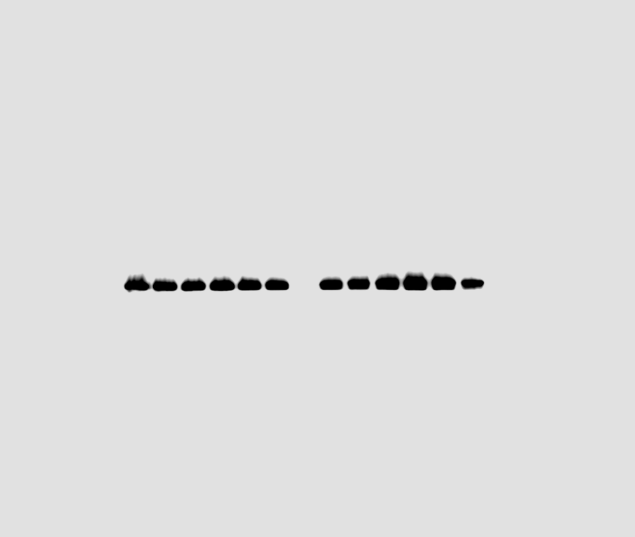


3.GAPDH


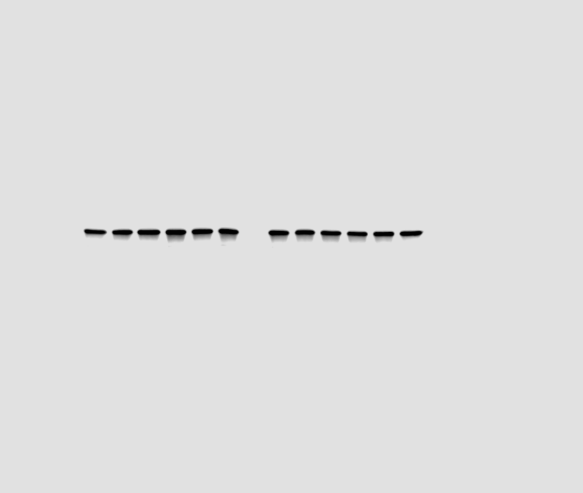

Supplement: Supplementary file 3 — Supplementary Material 3 [file 40659_2026_688_MOESM3_ESM.docx]
