# Supplementary material for: OGDH mediates α-ketoglutarate-induced follicular development and antioxidative response by interacting with CAT/SOD2
Source: Biol Res. 2026 Apr 10;59:33. doi: 10.1186/s40659-026-00688-9 (PMC13200353; doi:10.1186/s40659-026-00688-9)

4C

CASP8 (pcDNA3.1/pcDNA3.1-OGDH)


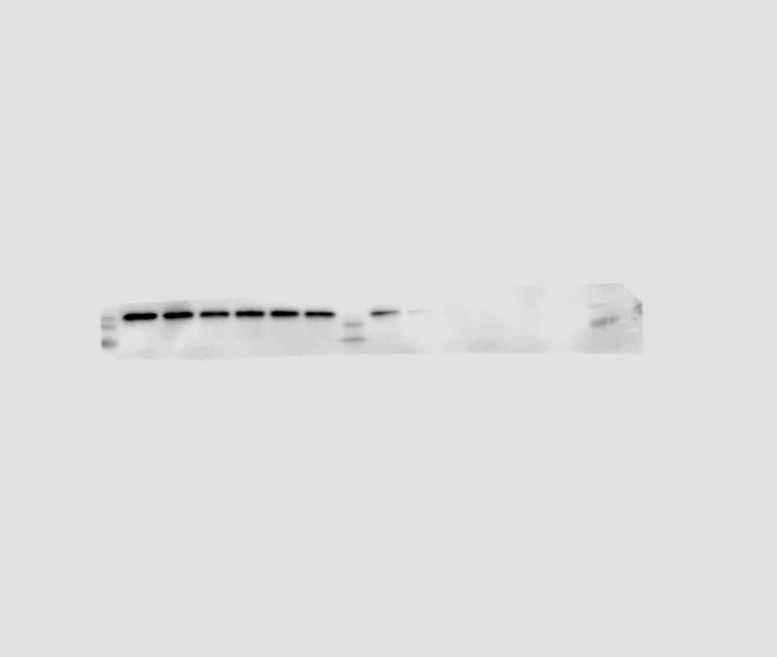


CASP8-(si-NC/si-OGDH)


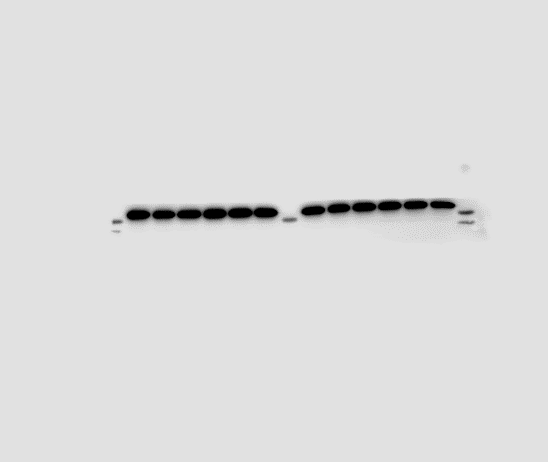


GAPDH-PCDNA3.1


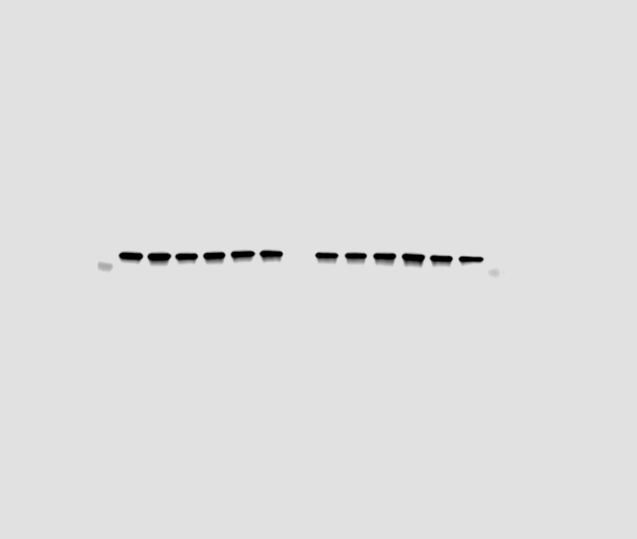


GAPDH-si


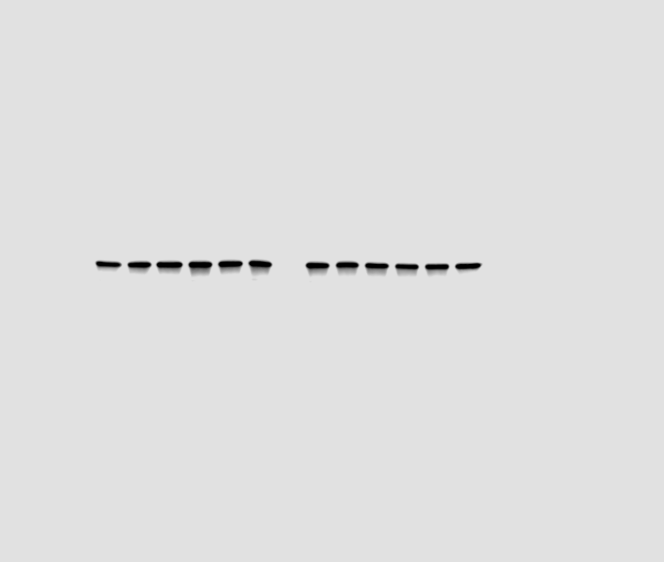


P53(pcDNA3.1/pcDNA3.1-OGDH)


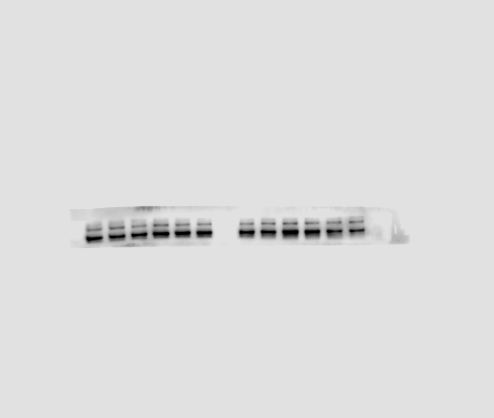


P53(si-NC/si-OGDH)


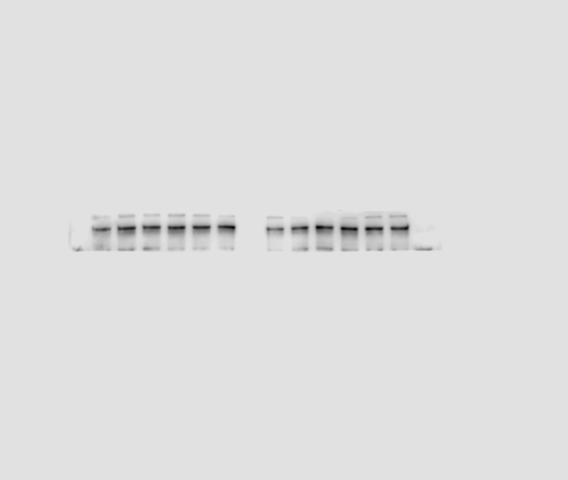


4F

CAT (left side pcDNA3.1/pcDNA3.1-OGDH, right side si-NC/si-OGDH)





SOD2 (left side pcDNA3.1/pcDNA3.1-OGDH, right side si-NC/si-OGDH)





GAPDH





4G

Input/OGDH/IgG


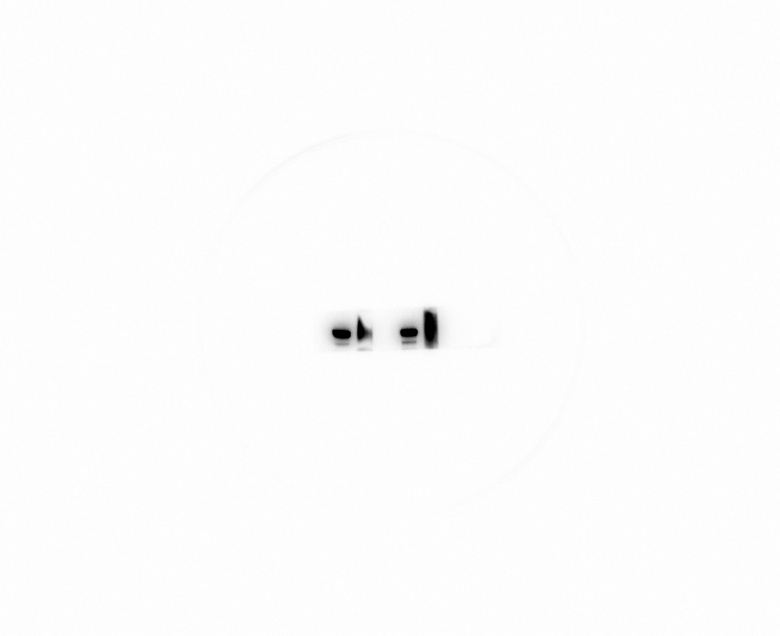


CAT(Input/OGDH/IgG)


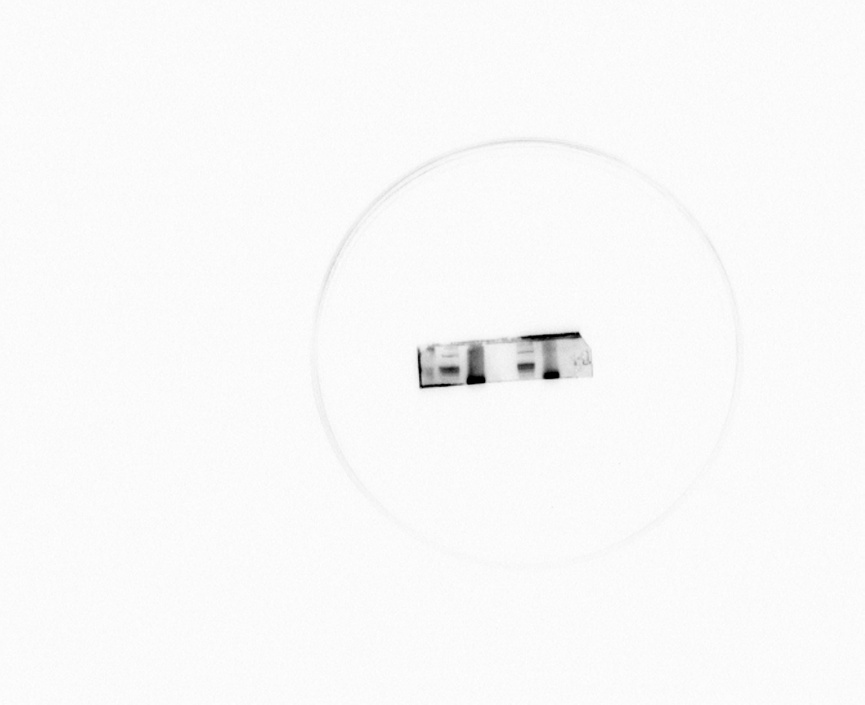


SOD2(Input/OGDH/IgG)


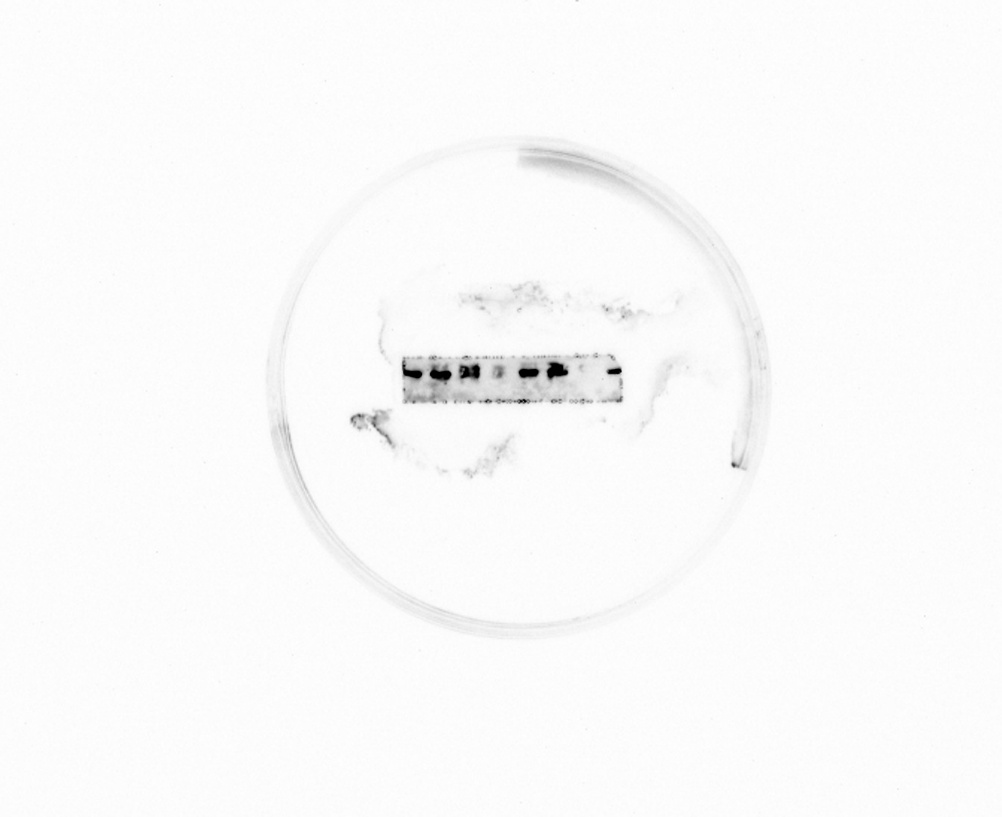


SOD1(Input/OGDH/IgG)


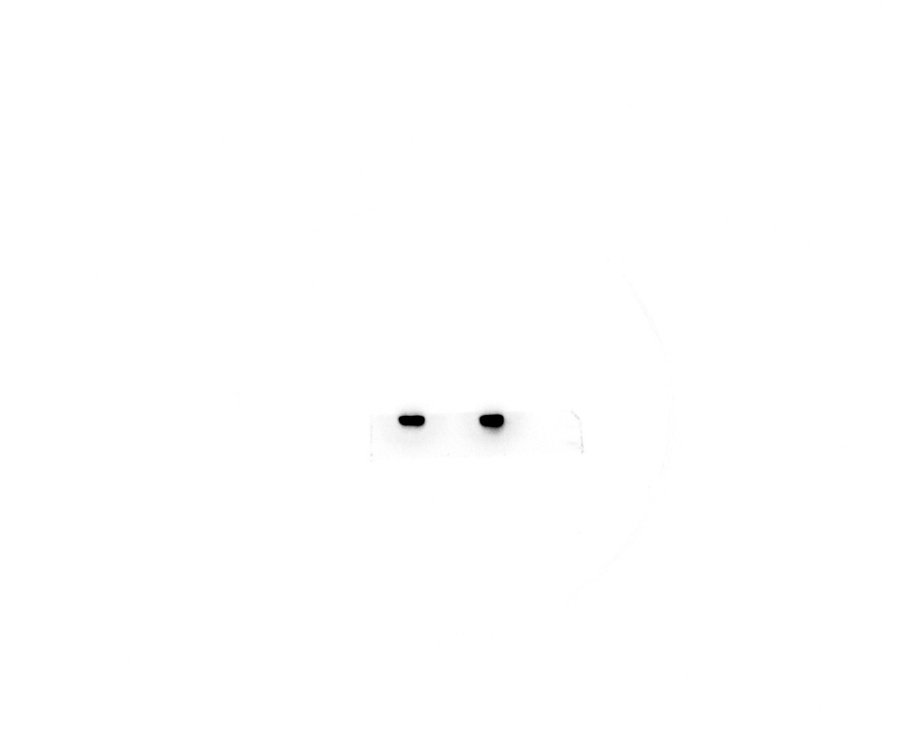


GAPDH(Input/OGDH/IgG)


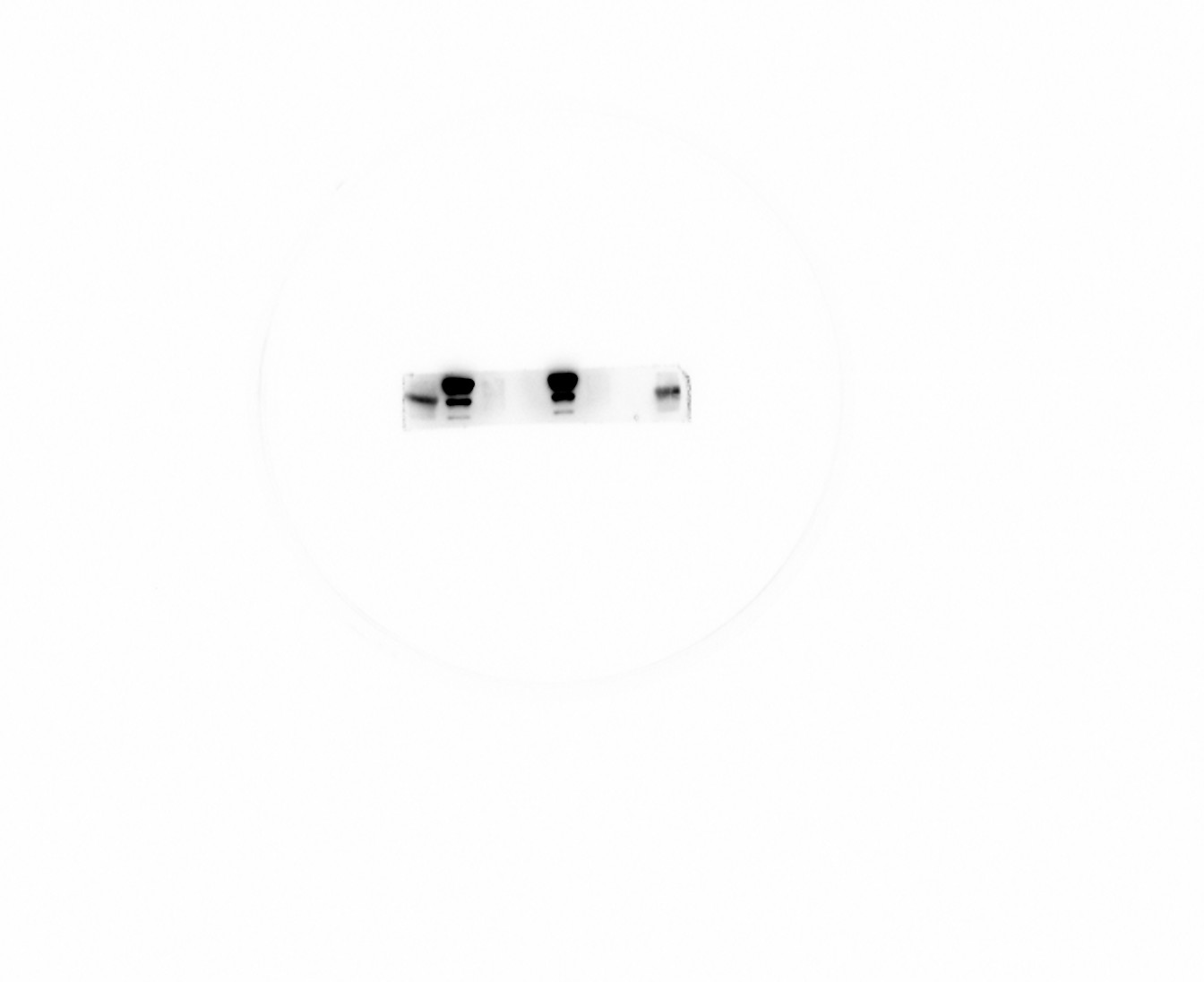

Supplement: Supplementary file 4 — Supplementary Material 4 [file 40659_2026_688_MOESM4_ESM.docx]
