# Supplementary figures and images for: OGDH mediates α-ketoglutarate-induced follicular development and antioxidative response by interacting with CAT/SOD2
Source: Biol Res. 2026 Apr 10;59:33. doi: 10.1186/s40659-026-00688-9 (PMC13200353; doi:10.1186/s40659-026-00688-9)

OGDH(small/middle/large)


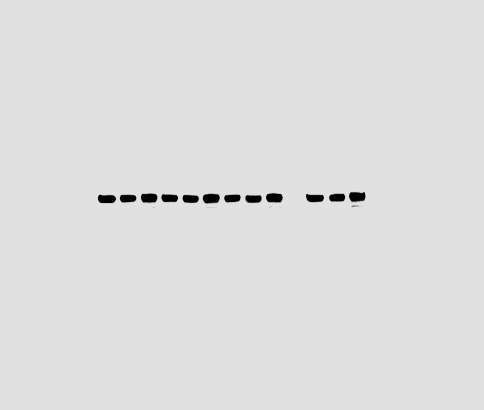


Tubulin


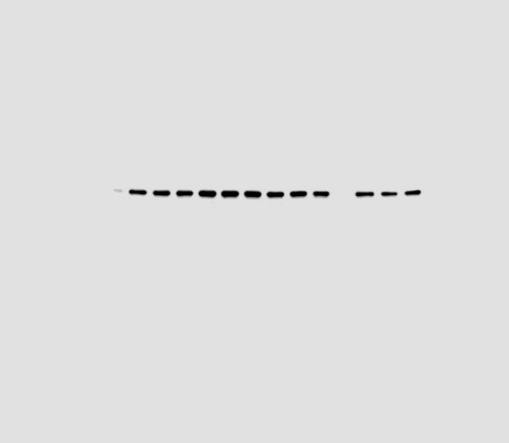

Supplement: Supplementary file 5 — Supplementary Material 5 [file 40659_2026_688_MOESM5_ESM.docx]
