# Supplementary material for: OGDH mediates α-ketoglutarate-induced follicular development and antioxidative response by interacting with CAT/SOD2
Source: Biol Res. 2026 Apr 10;59:33. doi: 10.1186/s40659-026-00688-9 (PMC13200353; doi:10.1186/s40659-026-00688-9)

6B

OGDH（left side LV-NC/LV-OGDH, right side sh-NC/sh-OGDH）


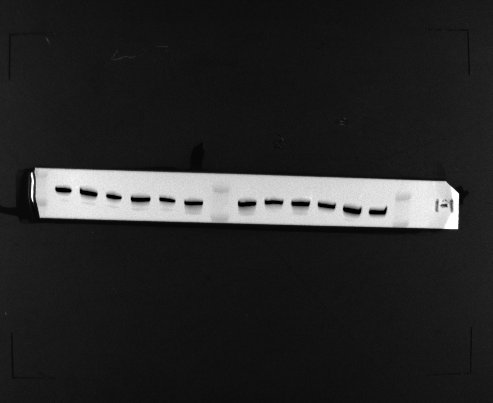


TUBULIN


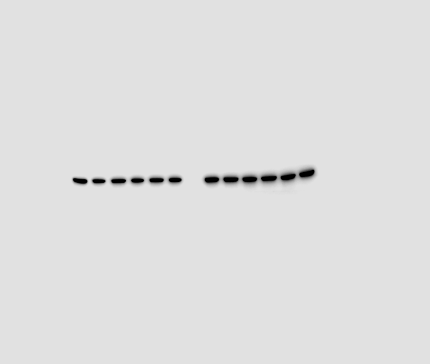


6I

CAS3（left side LV-NC/LV-OGDH, right side sh-NC/sh-OGDH）


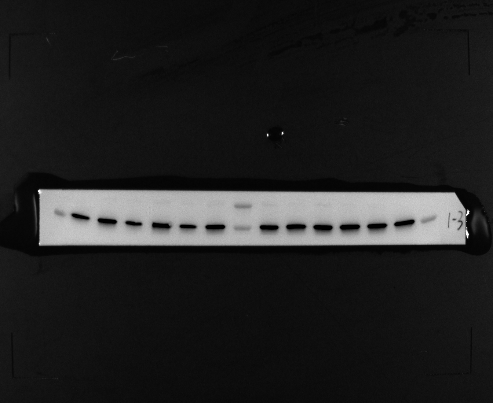


CAT（left side LV-NC/LV-OGDH, right side sh-NC/sh-OGDH）


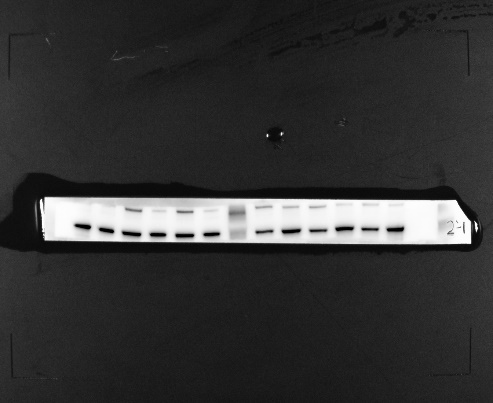


P53 （left side LV-NC/LV-OGDH, right side sh-NC/sh-OGDH）


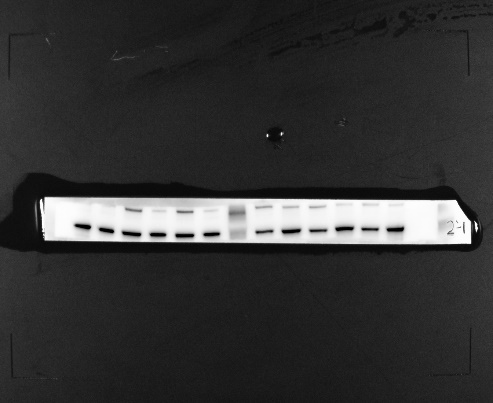


PCNA （left side LV-NC/LV-OGDH, right side sh-NC/sh-OGDH）


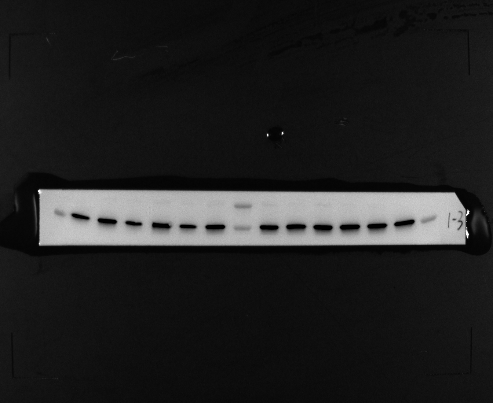


SOD2 （left side LV-NC/LV-OGDH, right side sh-NC/sh-OGDH）


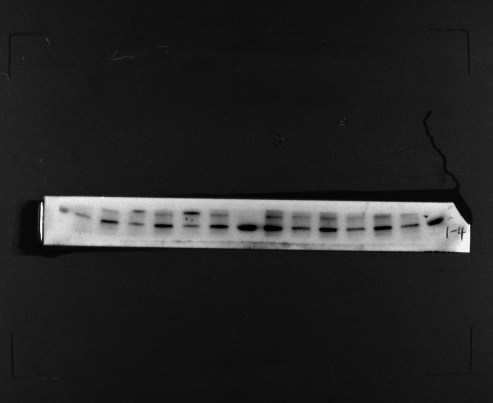


TUBULIN


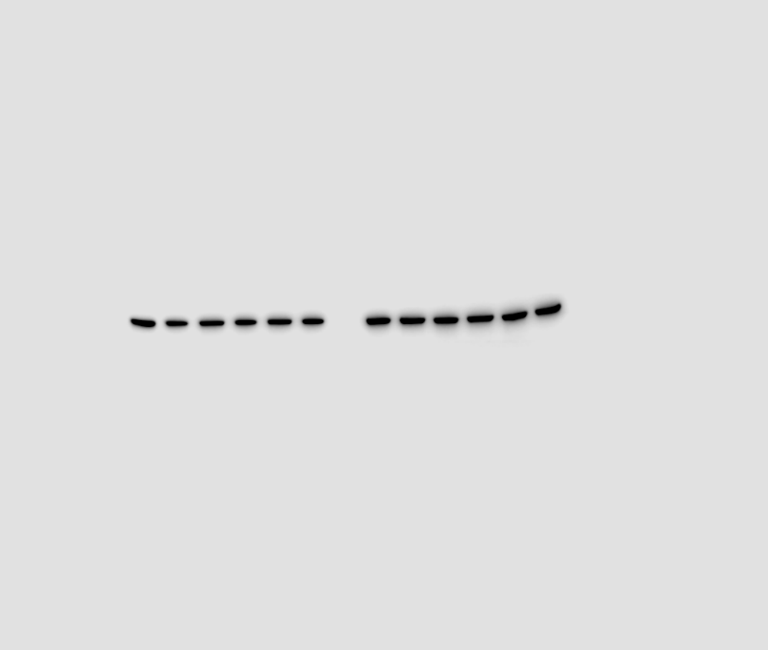

Supplement: Supplementary file 6 — Supplementary Material 6 [file 40659_2026_688_MOESM6_ESM.docx]
